# Talimogene Laherparepvec treatment for mucosal melanoma with metastatic lymph node metastasis: case report

**DOI:** 10.3389/fonc.2025.1647603

**Published:** 2025-10-15

**Authors:** Arnar B. Ingason, Venkatesa P. Muruganandam, Ikechukwu Chidobem, Jessica Cintolo-Gonzalez, Hibba tul Rehman

**Affiliations:** ^1^ Department of Surgical Oncology, University of Vermont Larner College of Medicine, Burlington, VT, United States; ^2^ Department of Medical Oncology, University of Vermont Larner College of Medicine, Burlington, VT, United States

**Keywords:** mucosal melanoma, malignant melanoma, T-VEC, immunotherapy, lymphatic metastasis

## Abstract

Mucosal melanoma is a rare and aggressive subtype of melanoma, accounting for approximately 1% of all melanoma cases. Of those, only 4% occur primarily in the vagina. Talimogene Laherparepvec (T-VEC) is a biopharmaceutical medication that is used to treat unresectable malignant melanoma. However, the clinical trials for this treatment option excluded patients with mucosal melanomas. Here we report a case of an 85-year-old female with vaginal melanoma with regional lymph node metastasis that had complete durable response to combined T-VEC and immune checkpoint inhibition treatment. This is, to our best knowledge, the first reported case of T-VEC treatment for vaginal melanoma. It supports findings from case reports demonstrating good response to T-VEC in mucosal melanomas of the urethra, maxillary sinus, and the soft and hard palate. Prospective studies assessing the efficacy of T-VEC in treating mucosal melanoma are needed to confirm these findings.

## Introduction

Mucosal melanoma is a rare and aggressive subtype of melanoma, accounting for approximately 1% of all melanoma cases in the United States (1). Due to its rarity and differing biology from cutaneous melanoma, mucosal melanomas are often excluded from clinical trials, or if included, remain represented in small numbers. There is a lack of specific treatment guidelines for mucosal melanoma, with treatment approach largely extrapolated from guidelines for cutaneous melanoma (2).

Talimogene Laherparepvec (T-VEC) is a biopharmaceutical medication that is used to treat unresectable malignant melanoma and is the first FDA-approved intralesional therapy for melanoma. It is a bioengineered herpes simplex virus 1 that infects both healthy and cancer cells. However, the virus lacks infected cell protein 34.5 (ICP34.5), a protein that inhibits protein kinase R, and allows herpes viruses to hijack the cellular translational machinery of the cell and replicate (3). Therefore, viruses without the ICP34.5 protein are unable to replicate in healthy cells. However, in melanoma cancer cells, the protein kinase R pathway is typically disrupted, allowing the bioengineered virus to replicate in those cells. This ultimately leads to lysis of the cancer cells, releasing viral particles, tumor-presenting antigens, and danger-associated molecular patterns (DAMPs). This allows for continued infection of cancer cells as well as stimulating the patient’s immune response to the tumor, thereby potentially expanding the tumor debulking effect beyond the treated area. T-VEC also promotes macrophage colony-stimulating factor (GM-CSF) production in infected cells, further stimulating the local immune response (3). T-VEC is administered by local injection into the tumor.

The OPTiM trial compared T-VEC treatment to GM-CSF administration in patients with unresectable stage IIIB/IIIC/IV melanoma with cutaneous, subcutaneous, or nodal sites (4). The study showed durable response rate of 19% for T-VEC compared to 1% for GM-CSF, and a complete response rate of 17% versus 1% for GM-CSF. However, patients with mucosal melanoma were excluded from the trial, resulting in a lack of data estimating the efficiency of T-VEC in patients with mucosal melanoma. Herein, we describe a case of a patient with vaginal mucosal melanoma that was successfully treated utilizing T-VEC treatment with durable response.

## Case presentation

The patient is an 85-year-old female with past medical history of hypertension, coronary artery disease, hyperlipidemia, hypothyroidism, gastroesophageal reflux, osteoporosis, and right-sided sciatica who had initially been diagnosed with vaginal mucosal melanoma in 2018 following new onset of postmenopausal bleeding (**Table 1**). She underwent a PET scan that showed 3 foci of radiotracer uptake deep in the pelvis. Exam was consistent with a primary bleeding mass and in transit disease making her initial stage at least Stage IIIC. Genomic testing demonstrated an *NRAS* Q61L mutation.

**Table 1:** Patient and tumor characteristics.

History of primary diagnosis and medical history	
Gender, age	Female, 85 years
Staging of primary and lump node status	Mucosal melanoma of the vagina; 4mm; Ulceration UN; T4, N3c, M0, stage IIIC
Mutational profile	*BRAF* negative *c-KIT* negative *NRAS* positive
Adjuvant therapy	nivolumab pembrolizumab
Medical history	Hypertension Coronary artery disease Hyperlipidemia Hypothyroidism Gastroesophageal reflux Osteoporosis Sciatica
Family history	Melanoma (sister) Non-Hodgkin’s lymphoma (brother) Esophageal cancer (son)

**Figure 1:**
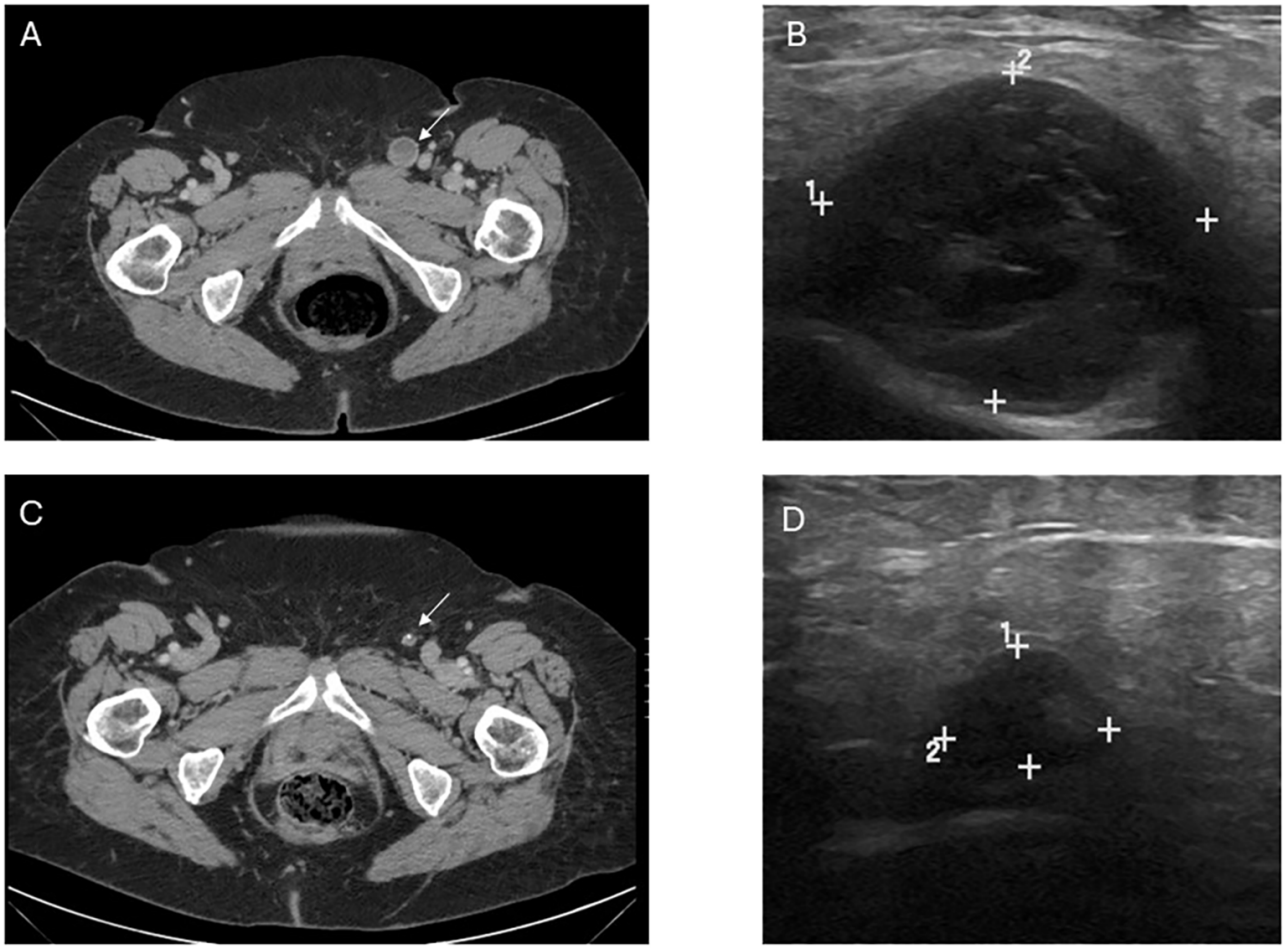
Image findings before and after T-VEC treatment. **(A)** Computed tomography (CT) demonstrating the abnormal lymph node consistent with metastatic melanoma prior to treatment. **(B)** Pretreatment ultrasound showing the enlarged, hypoechoic lymph node in the left inguinal region corresponding with the CT finding and measuring 2.3 x 2.1 x 2.7 cm. **(C)** Post-treatment CT demonstrating decrease in size and near resolution of lymph node, in which fine needle aspiration was unable to retrieve any viable melanoma. **(D)** Post-treatment US with significant decrease in size, measuring 1.2 x 0.5 x 0.7 cm though with some persistent abnormal morphology. Post-treatment size and morphology remained stable on axial and sonographic imaging after treatment discontinuation.

**Figure 2:**
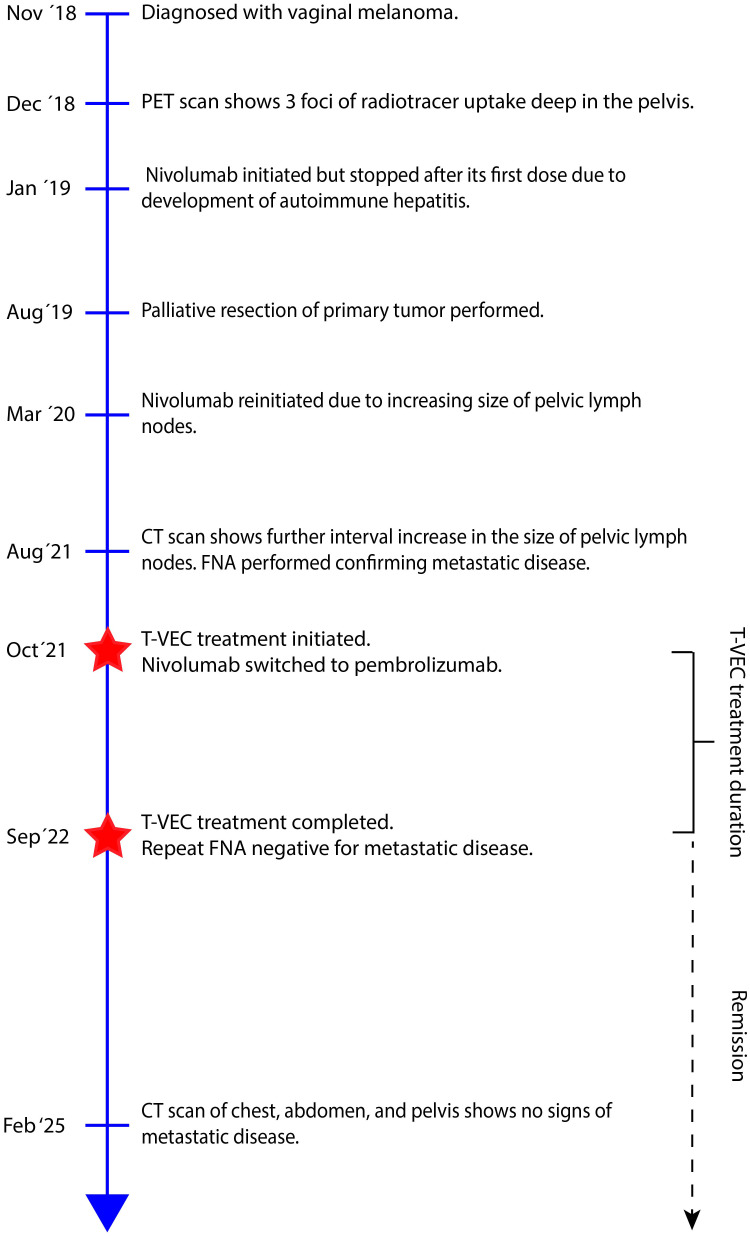
Timeline of patient’s clinical course from time of diagnosis to last scheduled follow-up. CT, Computed tomography; FNA, Final needle aspiration; PET, Positron emission tomography; T-VEC, Talimogene Laherparepvec.

She was started on immunotherapy with immune checkpoint inhibition (ICI) with nivolumab that was complicated by development of autoimmune hepatitis after its first dose in January 2019. This was treated with a steroid taper. She was offered pelvic exenteration but ultimately opted against such extensive surgery. In the following months, she developed worsening vaginal bleeding, causing significant lifestyle limitations, and she underwent palliative resection with R1 resection margins. Months later, her lymph nodes were noted to be enlarged and nivolumab therapy was restarted with palliative intent to maximize quality of life as patient was not amenable to extensive pelvic exenteration. She initially had partial response to the treatment as defined by the Immune Response Evaluation Criteria in Solid Tumors (iRECIST), without signs of hepatitis recurrence. However, a repeat computed tomography (CT) scan in 2021 showed progression of her disease, with a left inguinal lymph node noted to have increased from 0.8 cm to 2.0 cm in size. She therefore underwent a fine needle aspiration (FNA) confirming the diagnosis of metastatic malignant melanoma. Options for locoregional control including both surgical lymphadenectomy and intralesional therapy were discussed with the patient. She strongly desired to avoid the morbidity of surgery and was subsequently started on intralesional injections of T-VEC to the metastatic lymph node. She received T-VEC injections every 3 weeks over a 12-month time period with a total of 17 treatments. The initial dose was administered as 1 mL of 10^6 plaque-forming units (PFU). Subsequent doses were all full strength at 10^8 PFU with volume adjusted based on ultrasound measurements of the size of lymph node (ranged from 0.5–2 mL). Concomitantly, she was switched to pembrolizumab therapy so she could receive her ICI and T-VEC administrations on the same day. Repeat FNA of the treated lymph nodes after the completion of her T-VEC treatment showed no signs of residual metastatic disease in the lymph node. She continued in surveillance with clinical examinations, initially with frequent nodal ultrasound to supplement her axial imaging. Maintenance Pembrolizumab therapy was continued at 6-week intervals thereafter for 10 months. Of note, biopsy of the inguinal lymph node was negative though ctDNA was low positive at 0.06 mean tumor molecules/mL. Her latest CT scan of the chest, abdomen, and pelvis 2.5 years after cessation of T-VEC treatment and 19 months after discontinuing ICI did not show any signs of metastatic disease (**Figure 1**). A timeline of her clinical course is reported in **Figure 2**.

## Discussion

In recent years, the treatment of advanced stage melanoma has progressed rapidly. Unfortunately, the randomized controlled trials for these novel treatment options have generally excluded patients with mucosal melanomas, making treatment decisions in this population more challenging.

Mucosal melanoma carries a poor prognosis due to their concealed anatomical location, often resulting in delayed diagnosis and treatment. Unlike cutaneous melanoma, which is strongly linked to ultraviolet radiation exposure, mucosal melanoma arises on mucosal surfaces and follows a distinct pathogenesis. Within mucosal melanomas, 4% occur primarily in the vagina (5, 6). The vagina contains a unique mucosal environment, serving as a barrier to pathogens, while harboring tolerance to fetal antigens during pregnancy (7). While the generation of CD8+ T-memory cells is generally limited to secondary lymphoid tissue, such as the lymph nodes and spleen, the vagina has, uniquely, been demonstrated to harbor the ability to generate CD8+ T-cells within its mucosa (8). In fact, secondary lymph node tissue deficient mice have been demonstrated to yield efficient T-cell mediated immune response to an intravaginal herpes simplex virus 2 infection (9). This unique property may make vaginal melanomas particularly responsive to T-VEC, a genetically altered herpes simplex virus strain designed to augment local immune responses to melanoma. While T-VEC has been demonstrated to be a safe and efficient treatment for cutaneous melanoma metastatic to skin, subcutaneous, and nodal locations (4), there is limited data on its efficiency for metastatic mucosal melanoma.

The biology of mucosal melanomas differs considerably from cutaneous melanomas, with mucosal melanomas harboring a lower overall mutational burden and more chromosomal structural aberrations compared to cutaneous melanomas. The prevalence of mutations in *BRAF* are less common in mucosal melanomas meaning that a lower proportion of patients are eligible for targeted immunotherapy (10). Similarly, while RAS pathway mutations are present in some mucosal melanomas, there have not been approved effective therapies for those as of yet. Interestingly, while *c-Kit* mutations are rare in cutaneous melanoma, they have been reported in 14% of mucosal melanomas, making c-Kit inhibitors, such as imatinib, a possible target treatment for these tumors (11). A pooled analysis including 35 patients with *c-Kit* positive mucosal melanoma from 9 different studies demonstrated an overall response rate of 51% (11). However, this still leaves a large proportion of patients without a clinically actionable mutation. Increasing the challenge of treating mucosal melanoma further are findings that checkpoint inhibition treatment seems to be less efficient in treating mucosal melanoma than cutaneous melanoma (12). It has been hypothesized that the oncolytic treatment of T-VEC treatment may augment a patient’s response to checkpoint inhibition (13). While treatment with combination of T-VEC and pembrolizumab was not shown to significantly improve progression-free survival or overall survival compared with placebo-pembrolizumab in a randomized phase III clinical trial (14), there is good retrospective data to support the efficacy of T-VEC for patients who progress or recur after or while on immunotherapy (15, 16). Just as in the OPTiM trial, mucosal melanoma patients were excluded from this phase III trial, limiting the generalizability of its findings to the mucosal melanoma population.

In the current case, the patient had progression of her disease while on checkpoint inhibition, leading to her being started on T-VEC treatment and she was hesitant to receive dual checkpoint blockade with ipilimumab and nivolumab given prior history of ICI hepatitis. During her 12-month treatment with T-VEC, she was maintained on ICI with complete durable response. Given this, it is unclear whether this complete response would have been achieved if T-VEC had been administered as a monotherapy or whether it acted synergistically with her checkpoint inhibition treatment. A previous case report of a patient with a mucosal melanoma of the soft and hard palate demonstrated a complete local regression of her disease with T-VEC monotherapy (17). This patient had an underlying autoimmune disease precluding immunotherapy. Otherwise, the efficiency of T-VEC as a monotherapy for mucosal melanoma is largely unknown.

As reported herein, T-VEC may be a reasonable locoregional control option in patients who cannot tolerate or do not have adequate disease control with ICI in metastatic *c-Kit* negative mucosal melanoma. While surgery can provide locoregional control for patients, often extent of disease and/or surgical morbidity limits surgical options for patients with mucosal melanoma. This report adds to the limited data for use of T-VEC in mucosal melanoma by describing its unique use for primary vaginal mucosal melanoma metastatic to regional lymph nodes. Previous case reports have demonstrated good response to T-VEC therapy in patients with urethral, maxillary sinus, and oral melanomas (13, 17, 18). This is, to our best knowledge, the first reported case of T-VEC therapy for vaginal mucosal melanoma.

In conclusion, we report a patient with vaginal melanoma with lymph node metastasis that had complete durable response to combined T-VEC and ICI therapy. Further prospective studies or incorporation of mucosal melanoma patients into larger clinical trials should be considered to assess the efficacy of T-VEC treatment in mucosal melanoma to confirm these findings.

## Data Availability

The original contributions presented in the study are included in the article/Supplementary Material. Further inquiries can be directed to the corresponding author.

## References

[1] Sergi MC Filoni E Triggiano G Cazzato G Internò V Porta C. Mucosal melanoma: epidemiology, clinical features, and treatment. Curr Oncol Rep. (2023) 25:1247–58. doi: 10.1007/s11912-023-01453-x, PMID: 37773078 PMC10640506

[2] Swetter SM Johnson D Albertini MR Barker CA Bateni S Baumgartner J. NCCN guidelines^®^ Insights: melanoma: cutaneous, version 2.2024. J Natl Compr Canc Netw. (2024) 22:290–8. doi: 10.6004/jnccn.2024.0036, PMID: 39019054

[3] Kohlhapp FJ Kaufman HL. Molecular pathways: mechanism of action for talimogene laherparepvec, a new oncolytic virus immunotherapy. Clin Cancer Res. (2016) 22:1048–54. doi: 10.1158/1078-0432.CCR-15-2667, PMID: 26719429

[4] Andtbacka RHI Collichio F Harrington KJ Middleton MR Downey G Öhrling K. Final analyses of OPTiM: a randomized phase III trial of talimogene laherparepvec versus granulocyte-macrophage colony-stimulating factor in unresectable stage III-IV melanoma. J Immunother Cancer. (2019) 7:145. doi: 10.1186/s40425-019-0623-z, PMID: 31171039 PMC6554874

[5] Hou JY Baptiste C Hombalegowda RB Tergas AI Feldman R Jones NL. Vulvar and vaginal melanoma: A unique subclass of mucosal melanoma based on a comprehensive molecular analysis of 51 cases compared with 2253 cases of nongynecologic melanoma. Cancer. (2017) 123:1333–44. doi: 10.1002/cncr.30473, PMID: 28026870

[6] Chang AE Karnell LH Menck HR. The National Cancer Data Base report on cutaneous and noncutaneous melanoma: a summary of 84,836 cases from the past decade. The American College of Surgeons Commission on Cancer and the American Cancer Society. Cancer. (1998) 83:1664–78. doi: 10.1002/(SICI)1097-0142(19981015)83:8<1664::AID-CNCR23>3.0.CO;2-G, PMID: 9781962

[7] Zhou JZ Way SS Chen K. Immunology of the uterine and vaginal mucosae. Trends Immunol. (2018) 39:302–14. doi: 10.1016/j.it.2018.01.007, PMID: 29433961

[8] Wang Y Sui Y Kato S Hogg AE Steel JC Morris JC. Vaginal type-II mucosa is an inductive site for primary CD8^+^ T-cell mucosal immunity. Nat Commun. (2015) 6:6100. doi: 10.1038/ncomms7100, PMID: 25600442 PMC4348041

[9] Roth KL Bhavanam S Jiang H Gillgrass A Ho K Ferreira VH. Delayed but effective induction of mucosal memory immune responses against genital HSV-2 in the absence of secondary lymphoid organs. Mucosal Immunol. (2013) 6:56–68. doi: 10.1038/mi.2012.48, PMID: 22718264

[10] Hayward NK Wilmott JS Waddell N Johansson PA Field MA Nones K. Whole-genome landscapes of major melanoma subtypes. Nature. (2017) 545:175–80. doi: 10.1038/nature22071, PMID: 28467829

[11] Tacastacas JD Bray J Cohen YK Arbesman J Kim J Koon HB. Update on primary mucosal melanoma. J Am Acad Dermatol. (2014) 71:366–75. doi: 10.1016/j.jaad.2014.03.031, PMID: 24815565

[12] D’Angelo SP Larkin J Sosman JA Lebbé C Brady B Neyns B. Efficacy and safety of nivolumab alone or in combination with ipilimumab in patients with mucosal melanoma: A pooled analysis. J Clin Oncol. (2017) 35:226–35. doi: 10.1200/JCO.2016.67.9258, PMID: 28056206 PMC5559888

[13] Fröhlich A Hoffmann F Niebel D Egger E Kukuk GM Toma M. Talimogene laherparepvec in advanced mucosal melanoma of the urethra upon primary resistance on immune checkpoint inhibition: A case report. Front Oncol. (2020) 10:611. doi: 10.3389/fonc.2020.00611, PMID: 32457834 PMC7225290

[14] Chesney JA Ribas A Long GV Kirkwood JM Dummer R Puzanov I. Randomized, double-blind, placebo-controlled, global phase III trial of talimogene laherparepvec combined with pembrolizumab for advanced melanoma. J Clin Oncol. (2023) 41:528–40. doi: 10.1200/JCO.22.00343, PMID: 35998300 PMC9870217

[15] Carr MJ Sun J DePalo D Rothermel LD Song Y Straker RJ. Talimogene laherparepvec (T-VEC) for the treatment of advanced locoregional melanoma after failure of immunotherapy: an international multi-institutional experience. Ann Surg Oncol. (2022) 29:791–801. doi: 10.1245/s10434-021-10910-5, PMID: 34648098

[16] Balasubramanian A Salusti-Simpson M Callas P O’Neill C Rehman H Ahmed S. Factors associated with response to talimogene laherparepvec in the treatment of advanced melanoma. MI. (2024) 1:95–105. doi: 10.36922/mi.3445

[17] Chitnis SD Seim NB Kendra K. Local intralesional talimogene laherparepvec therapy with complete local response in oral palatine mucosal melanoma: a case report. J Med Case Rep. (2024) 18:257. doi: 10.1186/s13256-024-04554-8, PMID: 38778387 PMC11112787

[18] Drescher C Drexler K Hilber H Schmidt B Haferkamp S. Regression of mucosal melanoma following intralesional talimogene laherparepvec (T-VEC) injection in combination with immunotherapy. J Dtsch Dermatol Ges. (2019) 17:321–3. doi: 10.1111/ddg.13763, PMID: 30698924

